# Kalign – an accurate and fast multiple sequence alignment algorithm

**DOI:** 10.1186/1471-2105-6-298

**Published:** 2005-12-12

**Authors:** Timo Lassmann, Erik LL Sonnhammer

**Affiliations:** 1 Center for Genomics and Bioinformatics, Karolinska Institutet, Berzelius vag 35, S-17177 Stockholm, Sweden

## Abstract

**Background:**

The alignment of multiple protein sequences is a fundamental step in the analysis of biological data. It has traditionally been applied to analyzing protein families for conserved motifs, phylogeny, structural properties, and to improve sensitivity in homology searching. The availability of complete genome sequences has increased the demands on multiple sequence alignment (MSA) programs. Current MSA methods suffer from being either too inaccurate or too computationally expensive to be applied effectively in large-scale comparative genomics.

**Results:**

We developed Kalign, a method employing the Wu-Manber string-matching algorithm, to improve both the accuracy and speed of multiple sequence alignment. We compared the speed and accuracy of Kalign to other popular methods using Balibase, Prefab, and a new large test set. Kalign was as accurate as the best other methods on small alignments, but significantly more accurate when aligning large and distantly related sets of sequences. In our comparisons, Kalign was about 10 times faster than ClustalW and, depending on the alignment size, up to 50 times faster than popular iterative methods.

**Conclusion:**

Kalign is a fast and robust alignment method. It is especially well suited for the increasingly important task of aligning large numbers of sequences.

## Background

The alignment of multiple sequences is essential in the analysis of protein sequences [1]. In contrast to pairwise alignment, multiple sequence alignment (MSA) can reveal subtle similarities among large groups of proteins. Such information can be used in phylogenetic analysis [2], function prediction [3], HMM building [4], finding consensus sequences and in the identification of residues critical to function. Due to the importance of multiple sequence alignments in such a wide range of applications, a considerable amount of interest has been focused on improving the accuracy of MSA algorithms. Two basic modes of sequence alignment exist: global, i.e. over the entire length of the sequences [5], and local, in which only high-scoring areas are considered [6]. In general, database search algorithms use the local strategy [7-10], while most MSA algorithms use the global strategy. However, two noticeable exceptions are the two local MSA methods Poa [11] and Dialign [12,13]. Global methods tend to outperform local methods when sequences are related over their entire length [14], while local methods are superior in multiple domain cases when sequences may only share one common domain [15]. Since it is rarely known how sequences are related prior to the alignment, a method attempting to combine both local and global features was proposed by Notredame et al. [16]. Although this method produces good alignments [14,16,17] it suffers from being time consuming. Recently two new approaches, Mafft [18] and Muscle [19] were proposed. Both methods claim to produce alignments as accurate as those by T-Coffee while being considerably faster. The high accuracy is achieved by two iterative refinement strategies that are applied after an initial 'rough' alignment has been found. An important task for multiple sequences alignment algorithms in the future lies in the annotation and analysis of complete proteomes [20]. For an algorithm to be successful in such a setting it must produce high quality alignments consistently and, due to the volume of data, in a computationally feasible way. With these two goals in mind we developed Kalign, a global, progressive alignment method. We enhanced this approach by employing an approximate string-matching algorithm to calculate sequence distances and by incorporating local matches into the otherwise global alignment. We demonstrate that Kalign is well suited both in terms of speed and accuracy to deal with the challenges posed by large-scale comparative genomics.

## Algorithm

### Alignment strategy

The Kalign algorithm follows a strategy analogous to the standard progressive method for sequence alignment [21]. Pairwise distances are calculated, a guide tree is constructed and sequences/profiles are aligned in the order given by the tree. In contrast to existing methods, the Wu-Manber approximate string-matching algorithm [22] is used in the distance calculation and optionally in the dynamic programming used to align the profiles.

### Distance estimation and tree building

In progressive alignment the order in which sequences are aligned is crucial for accuracy. The goal is to align the most similar sequences first, to avoid errors in early stages of the alignment that cannot be corrected later on. This is normally accomplished by building a guide tree that dictates the order of pairwise alignments. The tree is typically built from a matrix of pairwise distances between all sequences, e.g. using the UPGMA [23] or the Neighbour-Joining [24] method. Performing all pairwise comparisons has quadratic complexity, and hence this step tends to dominate the running time of most progressive methods when aligning many sequences. Performing all pairwise alignments is slow but gives better distance estimates than approximate techniques, e.g. the k-tuple method employed optionally by ClustalW. In Kalign, sequence distances are estimated based on a novel method employing the Wu-Manber approximate string-matching algorithm. This strategy enables Kalign to estimate sequence distances more accurately but as fast as the k-tuple method. We will first discuss the Wu-Manber algorithm in some detail and then explain how it is incorporated into Kalign.

### Wu-Manber algorithm

The Wu-Manber algorithm is an extension to the exact Baeza-Yates-Gonnet algorithm [25] that allows string-matching with mismatches. The distance between two strings is measured by the Levenshtein edit distance. Two strings A and B have an edit distance d if A can be transformed into B by applying d mismatches, insertions or deletions. The Wu-Manber algorithm has a complexity of O(*tk*) where t is the length of the text string and *k *the number of errors allowed. Previously, protein sequences were commonly searched with patterns 2–3 long [7,9,10], and hence we choose to search with likewise short, three-residue patterns as well, allowing only one error. Since the maximum number of patterns under these setting is 8000 and only one error is allowed, all input sequences can be searched efficiently. A minor modification of the standard algorithm [22] allows us to search the sequences with 10 patterns at once, which speeds up this step. The benefit of using approximate string-matching when comparing protein sequences is obvious in two scenarios. Firstly, if sequences have an even degree of similarity along their entire length rather than patches of high identity, the standard k-tuple method fails to detect the similarity. For example, imagine comparing two sequences 'AAAAAA' and 'AALAAL'. Even though the sequences are 66% identical, the standard k-tuple method (with a word length of 3) finds no common patterns between the two sequences while the Wu-Manber algorithm finds 67 common patters ('AAA' matches sequence 1 with a mismatch and sequence 2 exactly, etc.). Similarly, sequences 'AAAAAA' and 'ALALAL' (50% sequence identity) share no exact patterns, yet 25 patterns with mismatches. Secondly, sequences that are less than 30% identical often share few, if any strings of length 3, so the resolution of the k-tuple methods starts to fail. However, shared mismatch patterns can still be readily found and enable the Wu-Manber algorithm to report meaningful distances even between highly divergent sequences. A drawback of using pattern matching for distance estimation is that many spurious matches are reported. Kalign overcomes this problem by considering the locality of the matches found as well as their total number. All matches add a score (16 if the pattern matches both sequences exactly, 8 if one has a mismatch, and 1 if both sequences have a mismatch) to the diagonal on the dynamic programming matrix on which they occur. In similar sequences one diagonal will get a very high score compared to the rest since many matches occur on the same diagonal. However, in distantly related sequences the distribution of scores is less clear and many diagonals will receive relatively low scores. Therefore, the sequence similarity in Kalign is defined as the sum of the three highest scoring diagonals found. By including three diagonals, only matches that are likely to be part of the final alignment are considered when estimating similarity, while many spurious matches are excluded. Although this method is marginally slower than the standard k-tuple counting method, it is much faster than performing all pairwise alignments. The pairwise similarity scores computed in this way are used by the UPGMA clustering method to construct the guide tree.

### Progressive alignment

At each internal node of the guide tree two sequences, two profiles or one sequence and one profile are aligned. Optionally, Kalign can use Wu-Manber matches as anchor points during the alignment phase, which requires two extra steps in addition to the dynamic programming. This option is intended to improve accuracy in cases of discontinuous alignments, e.g. if a domain has been inserted or deleted. It speeds up the dynamic programming, but the gain is cancelled out by the time to perform the extra steps.

### Dynamic programming

Kalign employs the global dynamic programming method using affine gap penalties as described in Durbin et al. [26]. Residues are assigned to three states (aligned, gap in sequence a, or gap in sequence b) which are interconnected by arrows representing transitions. The model has been modified to disallow a gap in one sequence to be immediately followed by a gap in the other sequences. Normally, three matrices of size (m + 1) (n + 1) are used to represent these states. When these 'state-matrices' are filled, the final cell contains the maximum alignment score, and a traceback procedure (requiring these matrices) is used to retrieve the actual alignment. To reduce the amount of memory required we combined two known strategies. Firstly, an additional matrix size (n + 1) (m + 1) is used to store which transition occurs in every cell of the dynamic programming matrix [27]. By doing so, the optimal alignment can be read off this 'trace-matrix' by looking at which transitions lead from the final cell to the first cell. Secondly, since the three 'state-matrices' are no longer needed to perform the traceback procedure, they can be reduced into one-dimensional arrays because each cell in the dynamic programming matrix only depends on values from the previous column. Thus, instead of using 3 ((n + 1) (m + 1)) memory we now only need 3 (n + 1) + ((n + 1) (m + 1)) memory. In practice, this translates into a ten-fold reduction in memory requirement. The reduction in memory reduces the number of cache-misses and makes the dynamic programming substantially faster. To our knowledge, the combination of these two methods has not been previously described. If the user wishes to use the option of including Wu-Manber matches as anchor points during the alignment phase, two additional steps are performed:

#### 1. Consistency check

The task of the consistency check is to sieve through the thousands of matches found between two sequences and find the largest set of matches that can be included into an alignment. For example: a pattern matching at position 100 in both sequences is inconsistent with a pattern that matches sequence A at position 20 and sequence B at position 150. Matches found in both sequences (or profiles) are plotted at the corresponding position into the dynamic programming matrix. Since it is possible for several patterns to match at the same position the number of matching patterns is recorded. The filled matrix is analogous to a homology matrix containing substitution scores in standard sequence alignment. A modified version of the Needleman-Wunsch algorithm is then used to find the path through the dynamic programming matrix that contains the highest number of consistent patters. Because we are interested in local matches here, no gap penalties are used. Finally, all shared matches that occur on too short diagonals are considered spurious and are excluded. We found that a cutoff of 22 residues long diagonals worked well.

#### 2. Updating of pattern match positions

Including matches in initial pairwise alignments involving regular sequences is relatively trivial. However, deeper into the guide tree, profiles are aligned both to each other and to sequences. The updating step adjusts the absolute position of matches found within sequences to their relative positions within the profiles generated by the dynamic programming step. For example, a match at position 50 in sequence A can end up in the 55^th ^column in a profile, if 5 gaps were inserted anywhere in sequence A before position 50. The matches initially 'tied' to individual sequences are thus assigned to matches within profiles and can be used in the next pairwise alignment step.

### Scoring system

Like other alignment programs based on dynamic programming, Kalign uses a substitution matrix and affine gap penalties. The choice of matrix and gap penalties has been the subject of previous studies [28]. The most commonly used substitution matrices are BLOSUM [29] and PAM [30]. A common idea is that similar sequences should be aligned with 'hard' matrices like PAM50 or BLOSUM80 while more distantly related sequences align better using 'soft' matrices like PAM250 or BLOSUM40. For instance, the commonly used program ClustalW adjusts the choice of substitution matrix accordingly. However, in agreement with Vogt et al. [28], we found no significant difference in alignment quality when using a soft matrix instead of a hard matrix on similar sequences. However, in the case of more distantly related sequences, hard matrices generally produce worse results than soft ones. Simply put, similar sequences are easy to align and the choice of substitution matrix does not noticeably affect the alignment quality. However, the correct alignment of dissimilar sequences requires using 'soft' matrices. Therefore we decided not to implement a complicated scheme that adjusts the choice of matrix but use a single soft matrix in all cases. We found little difference in alignment quality between using the BLOSUM50, PAM250 or GONNET250 [31] matrices and arbitrarily chose the GONNET250 matrix in combination with the parameters reported by Vogt et al. [28].

### Implementation

The Kalign algorithm was implemented in standard C.

## Methods

To compare Kalign to other MSA programs, the following test sets were used: the Balibase 2.01 [32,33] test set, Prefab 3.0 [19] and a new large test set.

### Balibase

The Balibase test set is a collection of alignments derived from structural databases or from manual alignments in the literature. We used the first five categories in Balibase version 2.01, containing 141 alignments. Each category represents some characteristic such as long or short sequences, high or low sequence identity, or large N/C terminal deletions or extensions. Reference alignments in Balibase contain only few, on average 10, and partial sequences. As noted before [17], this unnatural bias in the test set favours certain methods. We believe the real challenge lies in aligning large numbers of full-length sequences, which is currently not covered in Balibase. The diversity of the test set is further reduced because several sequences appear in more than one reference alignment.

### Prefab

Prefab exploits the abundance of pairwise structural alignment to create a multiple alignment test set. Each case in Prefab consists of a pairwise reference alignment and a set of sequences containing the two reference sequences plus 48 additional sequences that were obtained by querying a database with the reference sequences. To perform a test, the set of 50 sequences are aligned, the pairwise alignment of the two reference sequences is extracted from the resulting MSA, and is compared against the pairwise reference alignment. Prefab version 3 contains 1932 individual test cases, each based on a single pairwise alignment. Compared to the 141 test cases in Balibase this seems impressive, but each Balibase test case is an alignment of more than two sequences and in effect Balibase contains 8053 pairwise alignments.

**Figure 1:**
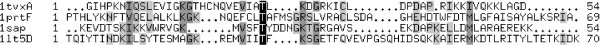
Balibase 2.01 reference alignment 1tvxA ref1 viewed by Belvu [37], showing conservation by "average similarity by BLOSUM62".

A drawback of both Balibase and Prefab is that sequence-based MSA methods strive to give evolutionarily motivated alignments that are inherently distinct from the structurally motivated reference alignments in the databases. In structural alignments, residues are assigned to the same column if they are considered structurally equivalent. In evolutionary alignments, aligned residues are thought to have originated from the same residue in a common ancestor. Although these two interpretations of the data often overlap, it is not always the case. Consider the Balibase alignment in Figure 1. Clearly, the sequences are very dissimilar from each other (the average sequence identity is 16%) and it is virtually impossible for any sequence-based alignment algorithm to even remotely reproduce this alignment. Even given structural information this alignment seems to be difficult since the corresponding reference alignment in Balibase 1.0 differs by 75% from the one shown in Figure 1. Comparing structural and sequence-based alignments can thus be problematic.

### Large testset

As Balibase and Prefab alignments are relatively small (10 and 50 sequences), we felt the need to examine performance on larger alignments. Since no real testset with large alignments exists, we were forced to use simulations to create this dataset. We used Rose version 1.3 [34], a program that simulates the evolution of sequences in a probabilistic fashion. Given a specified number of sequences and a target average evolutionary distance between them, Rose constructs a random phylogenetic tree, a random ancestor sequence at the root, and simulates evolution by applying substitutions, insertions, and deletions to create the sequences along the edges. As all the events in the history of the sequences are known, the true alignment is known and is recorded. Although some aspects of the simulation may be artificial, it is the only method that provides 100% knowledge of the true alignment. Obviously, alignments and sequences cannot be simulated perfectly by an evolutionary model. For example, two sequences modelled at a PAM distance of 200 might resemble real sequences at a PAM250 more closely than at PAM200. However, it is undeniable that also simulated sequences will become more and more difficult to align with increasing PAM distances. Alignment programs will align distant sequences differently, and based on this a meaningful and informative comparison between the programs can be made. A large test set such as this offers the opportunity to analyze the running times of alignment methods in depth.

### Quality measure

The alignment quality of each method was determined by calculating the sum-of-pairs score (SP) according to Thompson et al. [14]. This score reflects the percentage of correctly aligned residues determined by comparison to a reference alignment, and has little in common with the likewise called sum-of-pairs score often used as an objective function. We do not use the column score (CS) [14] as we feel this score is inadequate and does not reflect the biological correctness of alignments. For example, even if 99% of the sequences are correctly aligned, the column score can become zero due to a single misaligned sequence. In practice, the CS score gives lower accuracies than the SP score, but the ranking of the methods remains the same (results not shown).

### Alignment programs

We compared our method Kalign (with the default parameters) to Mafft version 3.85 [18], Muscle version 3.0 [19], ClustalW version 1.83 [35], Dialign version 2.2.1 [13] and T-Coffee version 1.37 [16]. A comprehensive review of the individual programs and their options is beyond this paper. Unless otherwise stated, we used the programs tested here in their highest accuracy mode. In the case of Mafft, four different scripts govern whether Fast Fourier Transform (FFT) and iterative refinement are used or not. Our experience in using Mafft revealed only small differences in quality and speed between the scripts using FFT or not (results not shown). We used the FFTNSI script throughout because it is slightly faster than the corresponding NWNSI (lacking FFT) script. The Muscle program, similar to the NWNSI script of Mafft, was run using all of the available refinement options.

## Results and Discussion

### Balibase

The average sum-of-pairs score was calculated for each category in Balibase and the results are shown in Table 1. Importantly, only conserved blocks in the Balibase alignments were used for evaluation. On this test set, Kalign performs slightly worse on average than Mafft and Muscle. We do not believe that these differences are meaningful due to the small size of alignments (on average 10 sequences) in Balibase and due to the fact that there are so few of them (141). Unfortunately, some of the methods tested here were developed using Balibase making comparisons to other methods problematic. For example, both ClustalW and Muscle were trained on Balibase [36]. Given that we did not make an attempt to train on Balibase we believe Kalign performs considerably well in general, and especially well in category 2 and 3.

**Table 1:** Balibase results. Categories ("Cat.") refer to the five Balibase categories. Average 1 is the average sum-of-pairs score over all 141 alignments, while average 2 is the average across all five categories.

Method	CPU time (sec.)	Cat. 1	Cat. 2	Cat. 3	Cat. 4	Cat. 5	Average 1	Average 2
Kalign	27	0.85	0.92	0.79	0.88	0.92	0.86	0.87
ClustalW	75	0.85	0.93	0.75	0.85	0.86	0.86	0.85
Muscle	75	0.88	0.93	0.82	0.88	0.97	0.89	0.89
Mafft	100	0.86	0.92	0.78	0.91	0.96	0.88	0.89
Dialign	234	0.80	0.89	0.68	0.90	0.94	0.83	0.84
T-Coffee	1286	0.86	0.93	0.78	0.92	0.96	0.88	0.89

### Prefab

Due to the large number of test cases in Prefab we limited this analysis to ClustalW, Kalign, Muscle and Mafft. The results (Table 2) agree with the results obtained from Balibase, with Kalign being approximately as accurate as Muscle and Mafft. It is worth noting that in 644 out of the 1932 cases Kalign produced better alignments than Muscle and in 702 cases better alignments than Mafft. In 1238 alignments the difference in accuracy between Kalign and Muscle was less than 1%. Similarly, there were 1232 alignments in which the difference between Kalign and Mafft was less than 1%. Based on this we conclude that Muscle, Mafft and Kalign are equally accurate on Prefab, and that all three methods are more accurate than ClustalW. Kalign was between 4 and 7 times faster than the other methods in completing all 1932 alignments.

**Table 2:** Prefab results

Method	CPU time (sec.)	Average
Kalign	1743	0.63
ClustalW	12404	0.59
Muscle	8824	0.63
Mafft	7653	0.64

### Large testset

**Figure 2:**
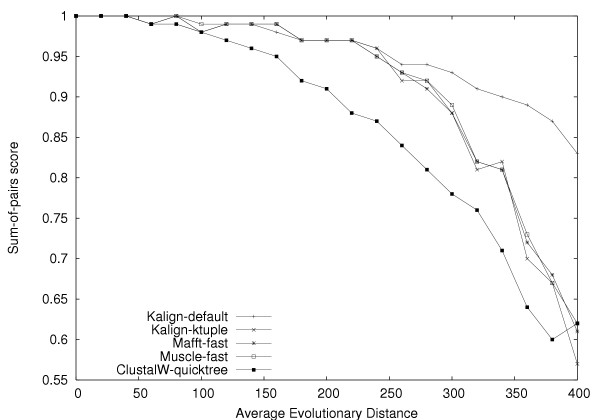
Analysis of the contribution to alignment accuracy made by different algorithmic variants. Kalign-default uses Wu-Manber approximate string matching, while Kalign-ktuple, Mafft-fast, Muscle-fast, and ClustalW-quicktree use exact k-tuple matching. The default Kalign Wu-Manber based algorithm becomes more accurate than other methods at high evolutionary distances. The alignments consisted of 50 simulated sequences.

**Figure 3:**
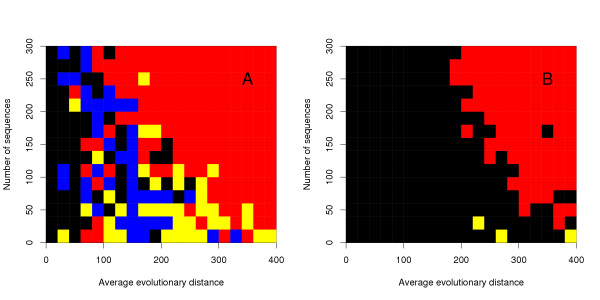
A 2D plot indicating in which situations different methods perform better on the large testset. The accuracy of the most accurate versions of Kalign, Muscle, and Mafft was measured for each combination of average evolutionary distance (in PAM units) and number of sequences. The cells were colored according to the most accurate program as: Kalign:red; Muscle:blue; Mafft:yellow. If there was a tie between two or more methods the cell is black. In (a) it is enough to win by the smallest margin, whereas in (b) the program must win by a margin of 2%. Up to 200 PAM no program stands out as a clear winner while above this distance Kalign dominates.

**Figure 4:**
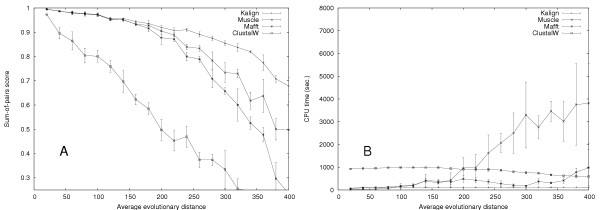
Plots of the accuracy (a) and speed (b) achieved by of Kalign, Mafft (FFTNSI), Muscle, and ClustalW on the large testset with increasing average evolutionary distance. The number of sequences (300) and the average sequence length (500 residues) are kept constant.

**Figure 5:**
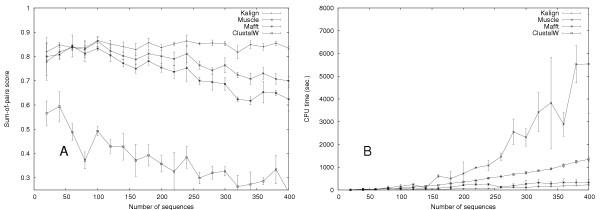
Plots of the accuracy (a) and speed (b) achieved by of Kalign, Mafft (FFTNSI), Muscle, and ClustalW on the large testset with increasing number of sequences. The evolutionary distance (300 PAM) and the average sequence length (500 residues) are kept constant.

To further evaluate the quality and speed of multiple alignment methods, we used our own large testset with up to 400 sequences per alignment, and up to an evolutionary distance of 400 PAM. T-Coffee and Dialign were excluded from these tests due to computational limitations. To investigate the effect of the Wu-Manber algorithm, we generated a series of alignments with Rose, each containing 50 sequences with an average evolutionary distance ranging from 0 – 400 (Figure 2). We tested the Kalign algorithm with the Wu-Manber string matching enabled (Kalign-default) and disabled (Kalign-ktuple). To make a fair comparison with other methods, we included versions of Mafft and Muscle with the iterative refinement disabled (FFTNSI and Muscle with the '-maxiters 1' option) and ClustalW run with the '-quicktree' option. In accordance with the results obtained on the Balibase and Prefab test set, ClustalW-quicktree is least accurate method. The alignment procedures used in Kalign-ktuple, Muscle-fast and Mafft-fast are comparable and as expected these methods are equally accurate. The default Kalign algorithm using the Wu-Manber algorithm becomes more accurate than the k-tuple based methods at high evolutionary distances. This confirms that the Wu-Manber method is superior to the k-tuple method traditionally used to estimate sequence distances and that both Mafft and Muscle could potentially benefit from using it. In order to examine the effects of evolutionary distance and number of sequences, we generated a test set containing 300 alignments. The evolutionary distance was varied in steps of 20 up to 400 and the number of sequences was gradually increased from 20 to 300 sequences. For each individual alignment in this test the winner, i.e. the program with the highest score, was determined (Figure 3). Kalign (shown in red) generally wins in difficult cases of high evolutionary distance and in cases with many sequences. The cases where Kalign does not win are alignments with few and highly similar sequences. Since these are relatively easy alignments the differences to Kalign are insignificant. As shown in Figure 2b, if a margin of 2% is required to call a winner, Kalign wins all the non-tied cases with only three exceptions that are won by Muscle. To further demonstrate the differences in accuracy and to analyze the running time in detail, we focused on one row and one column in Figure 3. The row was taken at 300 sequences, varying the evolutionary distance (Figure 4), and the column at an evolutionary distance of 300, varying the number of sequences (Figure 5). In contrast to Figure 3, the tests were repeated three times and the average scores are plotted with error bars. Figure 4a reveals that there is little difference in terms of quality between the methods at low evolutionary distances. However, at high evolutionary distances, Kalign is superior to Mafft and Muscle. In comparison to the other methods, ClustalW performs very poorly in this test. The analysis of the running times (Figure 4b), reveals a strong tendency of Mafft and Muscle to run increasingly slower with increasing evolutionary distances. In contrast, Kalign's complexity is not affected by evolutionary distance and is consistently faster than the other methods tested. ClustalW on the other hand, which starts off as the slowest method, becomes slightly faster with increased evolutionary distance and is in fact faster than both Mafft and Muscle at 400 PAM. The number of input sequences has a big effect on the running time of each method as the complexity of all alignment algorithms depend on it. Conversely, the more sequences that are used in an alignment, the better an alignment algorithm should perform. To our surprise, the quality of all methods except for Kalign decreased when the number of input sequences was increased (Figure 5a). The difference in alignment quality between Kalign and the next best method Muscle reaches 15% at 400 sequences. The analysis of running time versus increased number of sequences reveals a clear advantage of the Kalign algorithm over other methods (Figure 5b). Again Muscle is the slowest program; above 100 sequences it is on average four times slower than the second slowest program, ClustalW. Kalign takes 5 minutes to align 500 sequences while the same alignment takes 90 minutes using Muscle. Although we consider alignment quality to be the more important than speed, it is clearly advantageous in practice to have a fast alignment method.

## Conclusion

In this paper we present Kalign, a novel multiple sequence alignment algorithm based on Wu-Manber approximate pattern matching that combines high quality with high speed. Compared to existing programs, Kalign performed much more robustly when aligning large amounts of sequences or distant sequences in a large-scale benchmark of generated alignments. In terms of computational efficiency, Kalign is superior to the other methods, and readily aligns hundreds of sequences in minutes on a normal desktop computer. Coupled with the fact that Kalign gives very accurate alignments, this makes Kalign a very attractive overall method. The high accuracy of Kalign is due to the innovative use of the approximate Wu-Manber string-matching algorithm. This allows sequence distances to be accurately estimated even in difficult cases. Precise sequence distances generate good quality guide trees that, in turn, lead to good alignments. At the same time, Wu-Manber string-matching is very fast and dramatically cuts down the time required for the distance estimation step that dominates the running time of most alignment programs. The strategy detailed here can, in principle, be applied to any other progressive alignment method. Even when disregarding the results on the new large testset, Kalign's performance on Balibase and Prefab is impressive especially when considering that unlike other methods Kalign was not trained on either test set, and that other methods with similar performance are much slower.

## Availability and requirements

The Kalign program and a Kalign server are freely available at  or by request from the authors.

## Authors' contributions

TL had the idea of using a fuzzy-string matching algorithm in multiple sequence alignments, implemented the method and carried out the evaluation. ELLS supervised the work. All authors read and approved the final manuscript.
